# Association of red blood cell and platelet transfusions with persistent inflammation, immunosuppression, and catabolism syndrome in critically ill patients

**DOI:** 10.1038/s41598-021-04327-z

**Published:** 2022-01-12

**Authors:** Ginga Suzuki, Ryo Ichibayashi, Yuka Masuyama, Saki Yamamoto, Hibiki Serizawa, Yoshimi Nakamichi, Masayuki Watanabe, Mitsuru Honda

**Affiliations:** Critical Care Center, Toho University Omori Medical Center, Tokyo, Japan

**Keywords:** Immunology, Health care

## Abstract

The objective of this single-center retrospective cohort study was to investigate the relationship between blood transfusion and persistent inflammation, immunosuppression, and catabolism syndrome (PIICS). The study was conducted at the Critical Care Center at Toho University Omori Medical Center, Japan. We included 391 patients in the PIICS group (hospitalization for > 15 days, C-reactive protein > 3.0 mg/dL or albumin < 3.0 mg/dL or lymph < 800/μL on day 14) and 762 patients in the non-PIICS group (hospitalization for > 15 days and not meeting the PIICS criteria). We performed univariate and multivariate logistic regression analyses using PIICS as the objective variable and red blood cell (RBC) or fresh frozen plasma or platelet (PLT) transfusion and other confounding factors as explanatory variables. In addition, we conducted a sensitivity analysis using propensity score matching analysis. The multivariate and propensity score analyses showed that RBC and PLT transfusions were significantly associated with PIICS. This is the first study to report an association between RBC and PLT transfusions and PIICS. Our findings have contributed to better understanding the risk factors of PIICS and suggest that physicians should consider the risk of PIICS occurrence when administering blood transfusions in intensive care unit (ICU) patients.

## Introduction

Persistent inflammation, immunosuppression, and catabolism syndrome (PIICS) has been recently described in critically ill adults and has since been receiving increasing attention. PIICS refers to a condition in which a serious condition persists after intensive care in the acute phase. PIICS is mainly characterized by persistent inflammation, which is accompanied by immunosuppression and catabolism^1–3^ and often affects patients in the intensive care unit (ICU) and may affect their prognosis. Once PIICS develops, the patient's immunity weakens, activities of daily living worsen, and eventually, long-term mortality increases^1–3^. Therefore, it is imperative to predict and diagnose PIICS as early as possible. The original diagnostic criteria were length of hospital stay > 14 days, C-reactive protein (CRP) > 0.15 mg/dL, lymphocyte count < 800/μL, weight loss > 10% or body mass index < 18 during hospital stay, creatinine height index < 80%, albumin < 3.0 mg/dL, pre-albumin < 10 mg/dL, and retinol-binding protein < 10 μg/dL^1–3^. However, in 2020, Nakamura et al. proposed that CRP > 3.0 mg/dL was an additional appropriate criterion^4^. Nakamura et al. also identified the presence of disseminated intravascular coagulation (DIC) at admission as a factor associated with PIICS^5^.

Nevertheless, further evidence is required to identify the exact risk factors of PIICS. One potential risk factor of PIICS is blood transfusion; however, no study published to date has investigated the relationship between blood transfusion and PIICS. Trauma and surgery patients in the ICU often receive blood transfusions. In addition, anemia progresses in ICU patients without obvious bleeding^6–10^. The causative factors underlying anemia progression in ICU patients are not only acute bleeding but also blood dilution, dysregulation of iron metabolism, and blunting of erythropoietin response by inflammatory cytokines^8,9^. Blood products can cause transfusion-related immune modification (TRIM) that suppresses immunity^11–13^. Furthermore, the transfusion of red blood cell (RBC) products and platelets (PLT) are risk factors for nosocomial infections in various environments^14–17^. Moreover, Péju et al. reported that the administration of fresh frozen plasma (FFP) and PLT products in patients with sepsis increases the risk of infections after transfusion^18^. Péju et al. concluded that TRIM might also be present in FFP and PLT. Additionally, blood transfusion affects the immune system, especially in ICU patients, where blood transfusion is a frequently performed procedure.

We hypothesized that blood transfusion is associated with the onset of PIICS in ICU patients. Therefore, the aim of this study was to investigate the relationship between blood transfusions and PIICS.

## Methods

### Design, setting, and ethics approval

This is a single-center, retrospective cohort study, planned and conducted at the Critical Care Center, Toho University Omori Medical Center, in accordance with the Declaration of Helsinki as revised in 2013. It was approved by the Ethics Committee of the Toho University Omori Medical Center (approval number M20260). Due to the retrospective nature of the study, the need for written informed consent was waived.

### Subjects

The subjects were tertiary emergency patients admitted to the ICU between January 2019 and March 2021. Patients with acute deterioration in the hospital or those scheduled to undergo surgery were not included. The exclusion criteria were age < 18 years and death within 14 days of admission to the ICU. It should be noted that patients who were admitted to the ICU and moved to the general ward within 14 days were not excluded.

### Data collection

At this hospital, the profile, physical examination findings, laboratory findings, and treatment details of patients are recorded electronically. These electronic medical records were used as the data resource, and data retrieval began in July 2021. The following information was extracted from the medical records: patient profile (age, sex, height, and weight); primary disease; presence of sepsis (yes or no); surgical history; comorbidity (chronic heart failure, stroke, chronic obstructive pulmonary disease, diabetes mellitus, cancer, and Charlson Comorbidity Index [CCI] score^19^); acute physiology and chronic health evaluation (APACHE) II score; DIC (International Society on Thrombosis and Hemostasis [ISTH]-overt DIC)^20^ at admission; the presence of renal replacement therapy (RRT); received extracorporeal membrane oxygenation (ECMO; yes or no); duration of mechanical ventilation (MV); presence of shock (defined as a blood pressure less than 80 mmHg; yes or no); immunosuppressant use (yes or no); vasopressor use (yes or no); received blood transfusion (RBC, or FFP, or PLT; yes or no); the storage time of all transfusion products; and if patients were administered steroids (yes or no) on day 14. The following data from blood test results were extracted: CRP and albumin levels, lymphocyte count on admission and on day 14, creatinine (Cr) and hemoglobin (Hb) levels, PLT count, prothrombin time, fibrinogen, fibrin or fibrinogen degradation products (FDP), and lactate and glycated hemoglobin (HbA1c) levels. If there were no data available on day 14, the nearest values within days 12–16 were used instead.

### Definition of PIICS

Patients who required hospital stay (not ICU stay) for > 15 days and met the criteria of CRP > 3.0 mg/dL or albumin < 3.0 mg/dL or lymph < 800/μL on day 14 were assigned to the PIICS group^4,5^. Patients who required hospital stay for > 15 days and did not meet the PIICS criteria were assigned to the non-PIICS group.

### Outcome

The primary outcome was PIICS onset and the secondary outcome was 90-day mortality.

### Statistical analysis

We used the Kolmogorov–Smirnov test to test the normality assumption of our data; the variable was treated as following an abnormal distribution when the null hypothesis was rejected at 5% and as following a normal distribution when the null hypothesis was accepted. If the continuous variable had an abnormal distribution, it was expressed as the median (interquartile range) and tested using Mann–Whitney’s *U* test; if it was a normal distribution, it was expressed as mean ± standard deviation and tested using Student's *t* test. Categorical variables were expressed as percentages and tested using the chi-square test.

To analyze the factors related to the onset of PIICS, univariate analysis was performed with PIICS as the objective variable and with each of the following factors as explanatory variables: patient profile (age, sex, height, and weight); factors involved in the diagnosis of PIICS (CRP and albumin levels and lymphocyte count); factors reported as potential risk factors in previous studies (sepsis; Cr, Hb, and HbA1c levels, overt DIC, and APACHE II score)^5^; steroid use, underwent surgery, duration of mechanical ventilation, presence of shock, vasopressor use, and immunosuppressant use on day 14; and blood transfusion (RBC, FFP, PLT). Next, a multivariate logistic regression analysis was performed with PIICS as the objective variable and all the above variables as explanatory variables. Variance inflation factor was calculated for each factor, and if it was < 10, the factors were confirmed to have no multicollinearity. In addition, a receiver operating characteristic (ROC) curve was drawn to evaluate the diagnostic value of the volume of RBC transfused in predicting PIICS in ICU patients and the area under the curve (AUC) was calculated.

Next, sensitivity analysis using propensity score matching was performed. Propensity scores were calculated using a logistic regression model with the presence or absence of RBC transfusion as the objective variable and the following factors as explanatory variables that may have been considered in performing RBC transfusion: age; sex; height; weight; presence of sepsis; underwent surgery; CCI; Hb level; PLT counts; prothrombin time; fibrinogen, FDP, and lactate concentrations; APACHE II score; overt DIC; RRT; and ECMO. After calculating the propensity score, a caliper width of 0.2 was used for nearest neighbor matching. Multivariate logistic regression analysis was performed with a matched cohort using PIICS as the objective variable and the following factors as the explanatory variables: CRP, albumin, and Cr levels; lymphocyte counts, HbA1c levels; received steroids; duration of mechanical ventilation, presence of shock, vasopressor use, and immunosuppressant use on day 14 and blood transfusion (RBC, FFP, PLT). The explanatory factors used in the multivariate analysis were selected as a result of excluding the factors used to calculate the propensity score. Statistical analyses were performed using StatFlex^®^ version 7 (Artech Co., Ltd., Osaka, Japan). Statistical significance was set at p < 0.05.

## Results

**Figure 1:**
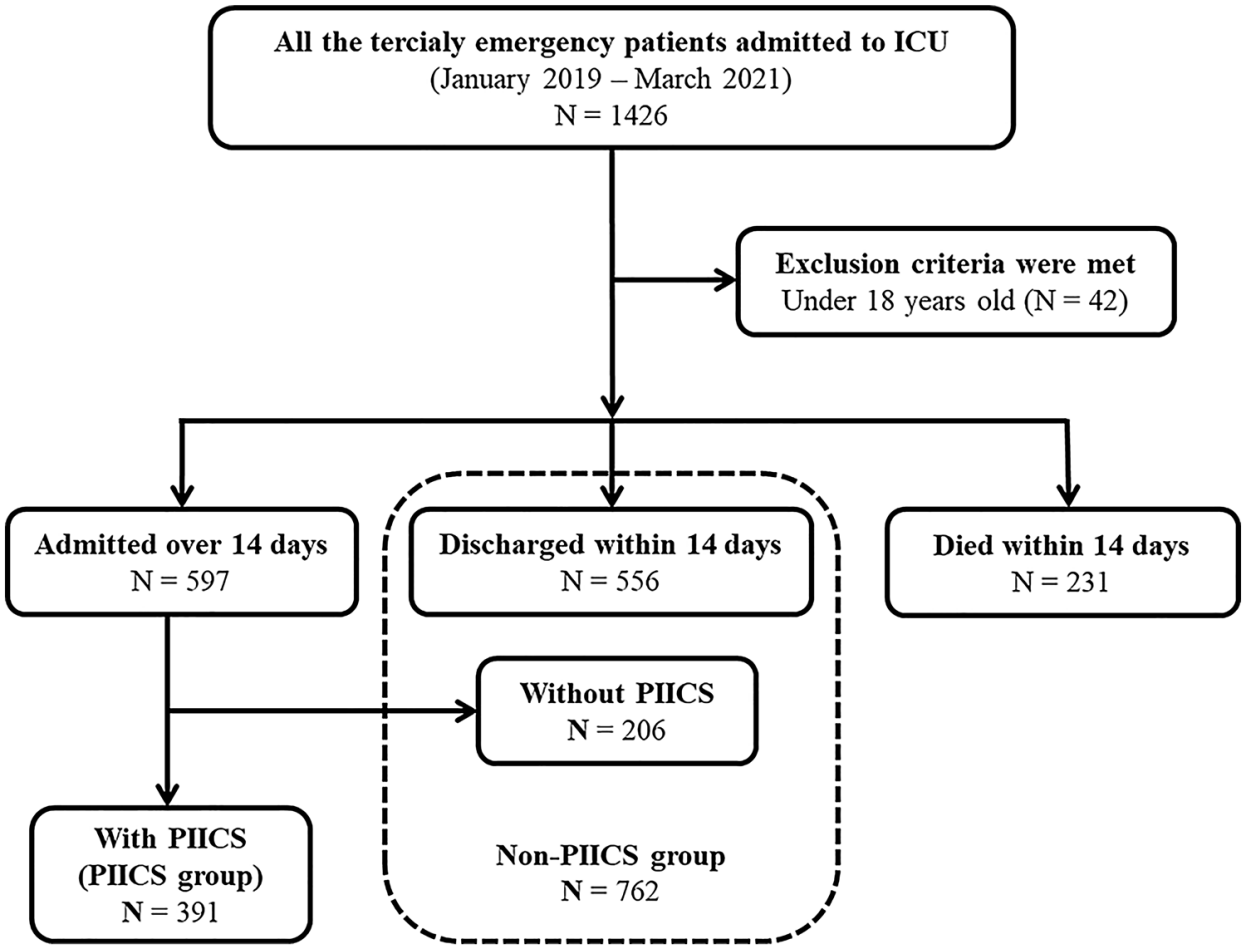
Chart showing the patient selection process. *PIICS* persistent inflammation, immunosuppression, and catabolism syndrome.

During the observation period, 1426 patients were admitted to the ICU from the emergency department. Forty-two patients were excluded because they were < 18 years old. Out of the remaining 1384 patients, 231 patients died within 14 days and were also excluded. As a result, 762 patients were grouped into the non-PIICS group and 391 patients into the PIICS group (Fig. 1).

Significant differences in most of the baseline factors were observed between the two groups (Table 1).

**Table 1:** Basic characteristics of overall and matched cohort.

Variable	Overall cohort			Matched cohort		
	Non-PIICS group (n = 762)	PIICS group (n = 391)	p	Non-PIICS group (n = 144)	PIICS group (n = 162)	p
Age, years	62.0 (30.0)	72.0 (22.0)	< 0.01	72.0 (23.6)	73.0 (20.0)	0.11
Male, n (%)	494 (65.2%)	260 (66.5%)	0.65	94 (65.3%)	97 (59.9%)	0.33
**Primary disease**			< 0.01			0.01
Cardiovascular, n (%)	115.0 (15.2)	64.0 (16.4)		24.0 (16.7)	20.0 (12.3)	
Respiratory, n (%)	56.0 (7.4)	26.0 (6.6)		11.0 (7.6)	7.0 (4.3)	
Digestive, n (%)	60.0 (7.9)	37.0 (9.5)		22.0 (15.3)	9.0 (5.6)	
Neurological, n (%)	76.0 (10.0)	80.0 (20.5)		13.0 (9.0)	39.0 (24.1)	
Metabolic, n (%)	22.0 (2.9)	11.0 (2.8)		3.0 (2.1)	5.0 (3.1)	
Sepsis, n (%)	24.0 (3.1%)	57.0 (14.6%)		10.0 (6.9%)	31.0 (19.1%)	
Abnormal body temperature, n (%)	33.0 (4.4)	14.0 (3.6)		10.0 (6.9)	6.0 (3.7)	
Cardiac arrest, n (%)	21.0 (2.8)	38.0 (9.7)		6.0 (4.2)	14.0 (8.6)	
Trauma, n (%)	154.0 (20.3)	37.0 (9.5)		25.0 (17.4)	17.0 (10.5)	
Others, n (%)	198.0 (26.1)	27.0 (7.4)		20.0 (13.9)	9.0 (5.6)	
Surgical procedure, n (%)	45.0 (5.9%)	110.0 (28.1%)	< 0.01	29 (20.1%)	54 (33.3%)	0.01
Charlson comorbidity index	0 (2.0)	1.0 (2.0)	< 0.01	1.0 (1.0)	1.0 (1.0)	0.55
Chronic heart failure, n (%)	46.0 (6.1)	29 (7.4)	0.38	11.0 (7.6)	14.0 (8.6)	
Stroke, n (%)	68.0 (9.0)	44.0 (11.3)	0.22	5.0 (3.5)	3.0 (1.9)	
COPD, n (%)	35.0 (4.6)	12.0 (3.1)	0.21	7.0 (4.9)	8.0 (4.9)	
Diabetes mellitus, n (%)	121.0 (16.0)	76.0 (19.4)	0.14	22.0 (15.3)	31.0 (19.1)	
Cancer, n (%)	78.0 (10.3)	51.0 (13.0)	0.16	23.0 (16.0)	21.0 (13.0)	
**Blood test on admission**						
C-reactive protein, mg/dL	0.1 (0.6)	0.8 (5.8)	< 0.01	0.2 (1.4)	0.9 (6.0)	< 0.01
Albumin, mg/dL	3.9 (0.9)	3.3 (1.1)	< 0.01	3.6 (0.9)	3.3 (1.3)	0.03
Creatinine, mg/mL	0.9 (0.5)	1.1 (1.0)	< 0.01	1.0 (0.9)	1.2 (1.0)	0.20
Lymphocyte counts, /μL	1849.0 (1876.8)	1185.0 (1986.9)	< 0.01	1439.5 (1945.2)	1139.5 (2136.9)	0.40
Hemoglobin, g/dL	13.4 (3.1)	12.7 (4.1)	< 0.01	11.3 (4.0)	12.3 (4.0)	0.02
Lactate, mmol/L	2.7 (3.2)	3.4 (5.3)	< 0.01	2.7 (3.5)	3.1 (5.1)	0.20
HbA1c, %	5.6 (0.7)	5.7 (1.0)	0.01	5.6 (0.7)	5.7 (1.0)	< 0.01
APACHE II score	14.0 (12.0)	20.0 (12.0)	< 0.01	17.0 (11.0)	20.0 (11.0)	0.01
Overt DIC, (%)	9.0 (1.2%)	21.05.4%)	< 0.01	6.0 (4.2%)	9.0 (5.6%)	0.57

*COPD* chronic obstructive pulmonary disease, *APACHE* acute physiologic and chronic health evaluation, *DIC* disseminated intravascular coagulation, *PIICS* persistent inflammation, immunosuppression, and catabolism syndrome.

There were also significant differences in almost all risk factors between the two groups for each factor on day 14 and when subsequently assessing the 90-day mortality rate (Table 2). There was no significant difference in storage time between the two groups for all products.

**Table 2:** Variables on day 14 and outcomes.

Variable	Non-PIICS group (n = 762)	PIICS group (n = 391)	p
**Blood test on day 14**			
C-reactive protein, mg/dL	0.5 (1.5)	3.6 (5.8)	< 0.01
Albumin, mg/dL	3.5 (0.7)	2.4 (0.7)	< 0.01
Lymphocyte counts, /μL	1432.9 (869.5)	1008.0 (688.2)	< 0.01
**Blood products on day 14**			
Red blood cell, n (%)	83.0 (10.9%)	193.0 (49.4%)	< 0.01
Storage time, day	12.0 (1.5)	12.0 (1.5)	0.38
Plasma, ml, n (%)	38.0 (5.0%)	110.0 (28.1%)	< 0.01
Storage time, day	269.5 (14.0)	266.0 (15.0)	0.67
Platelet, n (%)	6.0 (0.8%)	69.0 (17.6%)	< 0.01
Storage time, day	2.5 (0.5)	3.0 (0.5)	0.49
Steroids use on day 14, n (%)	36.0 (4.8%)	54.0 (13.9%)	< 0.01
Immunosuppressant use, n (%)	1.0 (0.1%)	5.0 (1.3%)	0.02
Vasopressor use on day 14, n (%)	100.0 (13.2)	208.0 (53.2)	< 0.01
Shock on day 14, n (%)	79.0 (10.4)	154.0 (39.4)	< 0.01
Duration of MV on day 14, day	0 (0)	1.0 (5.5)	< 0.01
RRT on day 14, n (%)	33.0 (4.4)	39.0 (10.0%)	< 0.01
ECMO on day 14, n (%)	3.0 (0.4%)	24.0 (6.1%)	< 0.01
90-day mortality	2.0 (0.3%)	57.0 (14.6%)	< 0.01

*PIICS* persistent inflammation, immunosuppression, and catabolism syndrome, *MV* mechanical ventilation, *RRT* renal replacement therapy, *ECMO* extracorporeal membrane oxygenation.

In the univariate analysis of the overall cohort, all factors except sex were significantly associated with PIICS. In addition, the multivariate analysis showed that age, sepsis, surgery, CRP, albumin level, lymphocyte count, Hb level, APACHE II score, duration of MV, and RBC and PLT transfusion were significantly associated with PIICS (Table 3).

**Table 3:** Univariate and multivariate analysis of PIICS in the overall cohort.

Parameters	Univariate analysis		Multivariate analysis		
	Odds ratio (95% CI)	p value	Odds ratio (95% CI)	p value	VIF
Age	1.032 (1.025–1.040)	< 0.01	1.019 (1.005–1.032)	0.01	2.08
Male	1.061 (0.820–1.372)	0.65	0.952 (0.579–1.566)	0.85	2.01
Sepsis	5.248 (3.202–8.601)	< 0.01	1.311 (1.008–1.707)	0.04	1.08
Surgical procedure	6.202 (4.270–9.009)	< 0.01	5.089 (2.793–9.271)	< 0.01	1.55
CCI	1.120 (1.028–1.220)	0.01	1.030 (0.906–1.171)	0.65	1.36
C-reactive protein	1.086 (1.062–1.109)	< 0.01	1.028 (1.000–1.058)	0.04	1.56
Albumin	0.367 (0.306–0.441)	< 0.01	0.471 (0.340–0.652)	< 0.01	2.39
Creatinine	1.108 (1.036–1.186)	< 0.01	0.984 (0.878–1.103)	0.79	1.60
Lymphocyte counts	1.000 (1.000–1.000)	< 0.01	1.000 (1.000–1.000)	< 0.01	1.22
HbA1c	1.125 (1.028–1.230)	0.01	1.027 (0.912–1.155)	0.66	1.15
Hemoglobin	0.902 (0.862–0.943)	< 0.01	1.118 (1.025–1.219)	0.01	2.27
APACHE II score	1.086 (1.069–1.104)	< 0.01	1.060 (1.030–1.091)	< 0.01	2.00
Overt DIC	4.749 (2.154–10.471)	< 0.01	2.218 (0.802–5.644)	0.13	1.08
Red blood cell	7.974 (5.895–10.786)	< 0.01	3.094 (1.822–5.253)	< 0.01	2.35
Plasma	7.458 (5.031–11.057)	< 0.01	0.727 (0.352–1.503)	0.39	2.40
Platelets	27.000 (11.606–62.815)	< 0.01	3.303 (1.134–9.625)	0.03	1.91
Duration of MV on day 14	1.347 (1.289–1.407)	< 0.01	1.280 (1.192–1.375)	< 0.01	2.93

*CCI* Charlson Comorbidity Index, *APACHE* acute physiologic and chronic health evaluation, *DIC* disseminated intravascular coagulation, *HbA1c* glycated hemoglobin, *MV* mechanical ventilation, *PIICS* persistent inflammation, immunosuppression, and catabolism syndrome, *VIF* variance inflation factor, *CI* confidence interval.

**Figure 2:**
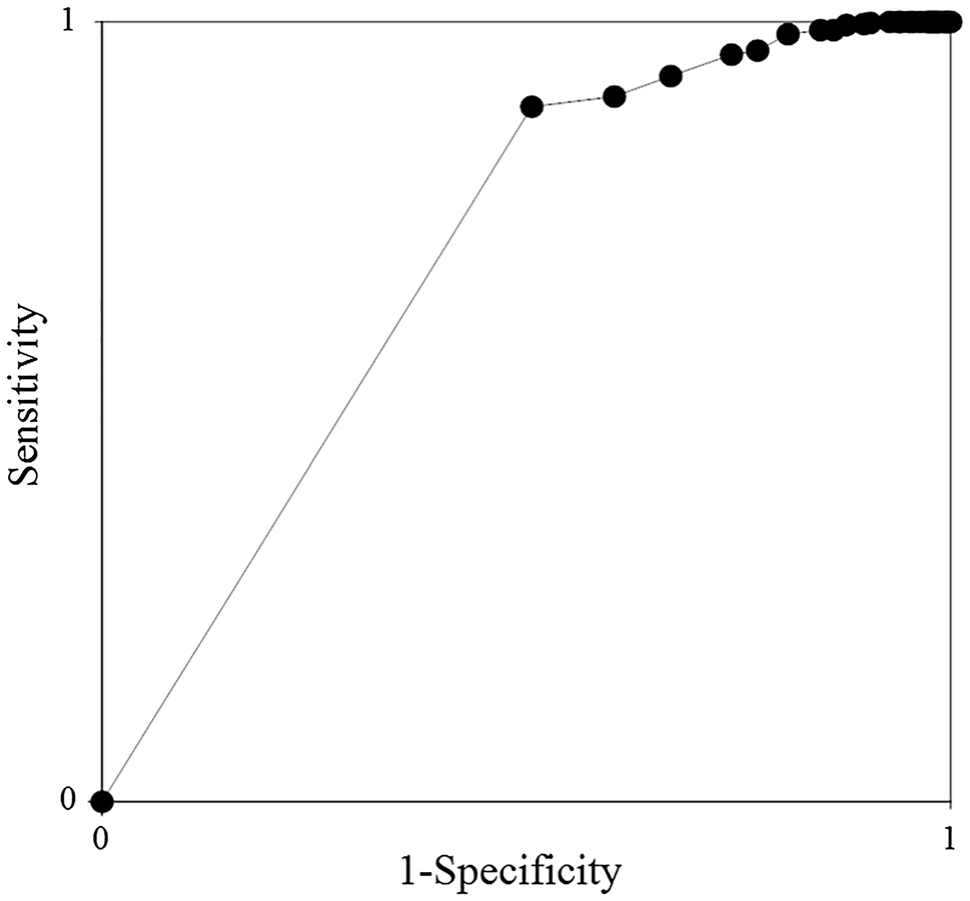
Receiver operating characteristic curve showing the diagnostic value of the volume of red blood cell transfused in predicting PIICS in ICU patients. The area under the curve is 0.70. *ROC* receiver operating characteristic, *PIICS* persistent inflammation, immunosuppression, and catabolism syndrome.

The ROC curve of PIICS for the RBC dose is shown in Fig. 2. The AUC was 0.70.

The RBC + PLT combination had a positive predictive value for PIICS of 93.1% (67/72), and the RBC + FFP + PLT combination had a positive predictive value for PIICS of 92.5% (62/67).

In the matched cohort, there were 144 patients in the non-PIICS group and 162 in the PIICS group. Compared with the patients in the non-PIICS group, those in the PIICS group had a significantly different distribution of primary diseases, higher incidence of surgery, higher CRP and Hb levels, HbA1c level, APACHE II scores, and lower albumin levels (Table 1). Fibrinogen and FDP were also significantly higher in the PIICS group (fibrinogen p = 0.02, FDP p = 0.01, not shown in table).

In the multivariate analysis with matched cohorts, CRP and HbA1c levels, duration of MV, and RBC and PLT transfusions were significantly associated with PIICS (Table 4).

**Table 4:** Multivariate analysis of PIICS in the matched cohort.

Parameters	Multivariate analysis		
	Odds ratio (95% CI)	p value	VIF
C-reactive protein	1.037 (1.004–1.080)	0.03	1.54
Albumin	0.746 ( 0.490–1.134)	0.17	1.39
Creatinine	0.994 (0.860–1.148)	0.93	1.19
Lymphocyte counts	1.000 (1.000–1.000)	0.43	1.08
HbA1c	1.392 (1.063–1.879)	0.02	1.08
Steroids	0.535 (0.180–1.594)	0.53	1.19
Red blood cell	3.186 (1.798–5.629)	< 0.01	2.84
Plasma	0.973 (0.906–1.045)	0.97	2.681
Platelets	1.077 (1.022–1.135)	< 0.01	1.78
Shock	2.107 (0.972–4.566)	0.06	1.70
immunosuppressant	1.882 (0.165–9.720)	0.73	1.05
Vasopressor	0.685 (0.298–1.570)	0.37	2.51
Duration of ventilation	1.263 (1.147–1.391)	< 0.01	2.24

*MV* mechanical ventilation, *PIICS* persistent inflammation, immunosuppression, and catabolism syndrome, *VIF* variance inflation factor, *CI* confidence interval, *HbA1c* glycated hemoglobin.

## Discussion

The present study results showed that PIICS is significantly associated with RBC and PLT transfusions. This result was consistent even after adjusting by propensity score matching. In addition to RBC and PLT transfusion, PIICS was associated with age, sepsis, surgery, CRP and albumin levels, lymphocyte count, Hb and HbA1c levels, APACHE II score, and duration of MV on day 14.

Nakamura et al. reported that age, sex, sepsis, albumin level, lymphocyte count, Hb and HbA1c levels, Cr level, and overt DIC were significantly associated with PIICS^5^. Thus, the results of the present study are consistent with their findings. However, in contrast to the report by Nakamura et al.^5^, which summarized the risk factors for patients admitted to the emergency ward and ICU regardless of in-hospital or outpatient settings; the present study included patients admitted to the ICU from the emergency department. The difference in patient cohorts between the two studies may explain the differences in findings. Specifically, the patients in this study were younger and mostly male. Furthermore, the number of patients with sepsis was smaller and most had a higher APACHE II score than those in the study reported by Nakamura et al.^5^. In addition, the overt DIC was surprisingly small in this study. Furthermore, a new analysis was conducted regarding blood transfusion, which may have caused the difference in the results.

It has been suggested that TRIM induces inflammation as well as immunosuppression via allogeneic mononuclear cells, leukocyte-derived soluble mediators, and soluble human leukocyte antigen peptide^11–13^. In addition, PLT-derived factors may cause immunosuppression, suggesting that PLT and residual-PLT in the RBC products may cause TRIM^11^. This study is the first study to investigate the relationship between the onset of PIICS and transfusion products. In addition, most of the transfusion products in this study were administered shortly after admission to the patients in the ICU. From the results of this study, it is hypothesized that the immunosuppressive effect and inflammation-inducing effect of blood transfusion last for about 14 days. It is also suggested that the administration of transfusion products could prolong high CRP levels and reduce lymphocyte counts. The mechanism of TRIM is not clear, but there may be a mechanism by which transfusion products reduce the number of lymphocytes. Critically ill patients, such as those admitted to the ICU, have anemia, thrombocytopenia, and are affected by various drugs. In such patients, the immunosuppressive effect of blood transfusions may be greatest. This study deals with the most severely ill patients in the PIICS studies, and TRIM may have been prominent. In the future, it is desirable to verify the effects of TRIM on critically ill patients in terms of cytokines and mediators.

Furthermore, RBC and PLT transfusions were associated with PIICS, suggesting that it would be important to consider transfusion criteria according to various diseases and stages in future studies.

This study has several limitations. First, the characteristics of the patient population may have influenced the results as this was a single-institution retrospective study; thus, the results cannot be generalized. Nevertheless, a relatively large number of samples were collected and analyzed. Second, the effects of confounding factors that were not considered in this study were not removed. Third, patient factors that required RBC transfusion may have influenced the outcome, and the effects were incompletely removed. However, as a countermeasure, propensity score matching analysis was performed. Fourth, there may be cases where the diagnosis of PIICS was inaccurate as there were no data available for day 14; in such cases, we included data from the nearest date. Fifth, It was not possible due to the nature of the study to collect donor information for each blood product administered. In addition, since all blood products used in this study were leukocyte-removed prior to storage, most leukocytes and platelets were removed. However, the presence of cytokines released from the cells of blood products cannot be ruled out, and data on cytokines could not be collected. These factors can also affect TRIM and PIICS.

In conclusion, this is the first study to report an association between RBC and PLT transfusions and PIICS. RBC + PLT transfusion showed a high positive predictive value for PIICS. However, it is suggested that PIICS does not increase even if FFP is added to the combination of RBC + PLT. Our findings have contributed to better understanding the risk factors of PIICS and suggest that physicians should consider the risk of occurrence of PIICS when administering blood transfusions in ICU patients.

## Data Availability

The datasets used and/or analyzed during the current study are available from the corresponding author on reasonable request.
